# Synergistic enhancement of efferocytosis and cholesterol efflux via macrophage biomimetic nanoparticle to attenuate atherosclerosis progression

**DOI:** 10.1016/j.bioactmat.2025.09.022

**Published:** 2025-09-19

**Authors:** Shiteng Cai, Jinfeng Gao, Xueyi Weng, Zhengmin Wang, Danwen Zheng, Qiaozi Wang, Qiyu Li, Chengzhi Han, Weiyan Li, Jing Chen, Yuyuan Fu, Yiwen Tan, Bohan Wei, Zhiqing Pang, Zheyong Huang, Yanan Song, Junbo Ge

**Affiliations:** a Department of Cardiology, Zhongshan Hospital, Fudan University, Shanghai Institute of Cardiovascular Diseases, China; b State Key Laboratory of Cardiovascular Diseases, Zhongshan Hospital, Fudan University, China; c NHC Key Laboratory of Ischemic Heart Diseases, China; d Key Laboratory of Viral Heart Diseases, Chinese Academy of Medical Sciences, China; e National Clinical Research Center for Interventional Medicine, Shanghai, China; f Institute of Biomedical Sciences, Fudan University, Shanghai, 20032, China; g School of Pharmacy Fudan University Key Laboratory of Smart Drug Delivery Ministry of Education, 826 Zhangheng Road, Shanghai, 200030, China

**Keywords:** Atherosclerosis, Efferocytosis, Cholesterol efflux, CD47-SIRPα axis, Biomimetic membranes

## Abstract

Atherosclerosis is the leading cause of myocardial infarction and stroke, which is characterized as a chronic inflammatory disease due to the aberrant accumulation of apoptotic cells in the necrotic core. Previous CD47-SIRPα checkpoint blockage strategies based on monoclonal antibodies or nanoparticles have shown significant pro-efferocytosis effects and thus improved the inflammatory microenvironment of plaque. However, apoptotic foam cells and concentrated cholesterol render plaque macrophages an overwhelming lipid burden, limiting the pro-efferocytosis effect of checkpoint blockade therapy in atherosclerosis. In this study, we fabricate a retinoic acid-loaded macrophage membrane-biomimetic liposome (R@MLP) to improve the efferocytosis ability of macrophages further. Mechanistically, the innate existence of SIRPα on the R@MLP would block the binding of CD47 on apoptotic cells with SIRPα on macrophages to realize the CD47-SIRPα inhibition. Consequently, engulfing retinoic acid in R@MLP would upregulate the expression of ABCA1 and ABCG1 of macrophages and enhance cholesterol efflux. In the mouse model of atherosclerosis, which benefited from the macrophage membrane, R@MLP showed ideal inflammation targeting ability to plaques and further reinforced the efferocytosis ability of macrophages. Ultimately, R@MLP shifted macrophages to the anti-inflammatory state and attenuated the progression of atherosclerosis. R@MLP synergizes checkpoint inhibition and cholesterol efflux to boost pro-efferocytosis therapy and presents a novel anti-inflammatory therapeutic strategy for atherosclerosis management.

## Highlights

• Retinoic acid-loaded macrophage membrane-biomimetic liposome R@MLP blocks CD47-SIRPα.
• Retinoic acid upregulates ABCA1/ABCG1, boosting macrophage cholesterol efflux.
• Biomimetic liposome targets plaques, boosting efferocytosis and M2 polarization.
• Retinoic acid-loaded macrophage membrane-biomimetic liposome slows atherosclerosis.

## Graphical abstract

**Figure:**
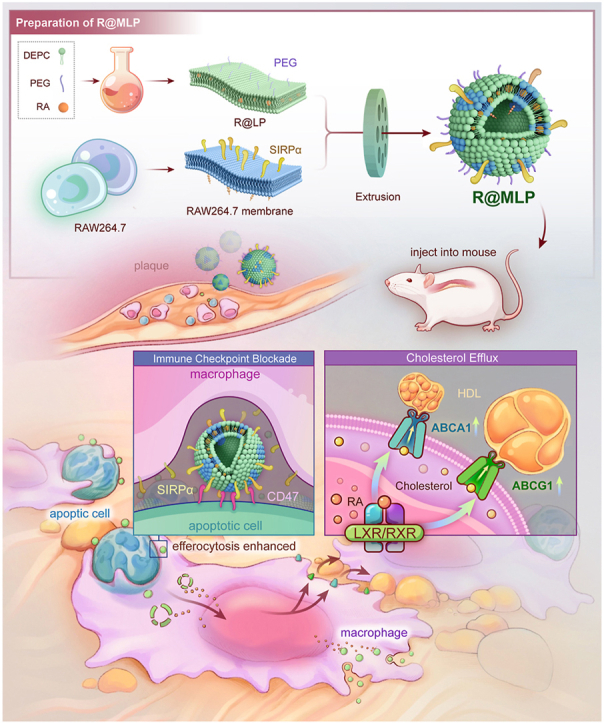

## 1 Introduction

Atherosclerosis is a chronic progressive inflammatory disease affecting medium and large arteries, characterized by the accumulation of lipids in the intima [1]. The instability and rupture of plaques lead to complications such as myocardial infarction and stroke [2]. Despite significant improvements in patient prognosis over the past few decades due to lipid-lowering therapies, particularly statins, the incidence of cardiovascular events remains alarmingly high [3]. As research into the mechanisms underlying the development and progression of atherosclerosis has become more apparent, attention has increasingly turned to the critical role of anti-inflammatory therapies in improving the prognosis of atherosclerosis [4]. Programmed cell clearance, also known as efferocytosis, is a physiological process through which apoptotic cells are cleared by macrophages, thereby maintaining homeostasis in the body [[5], [6], [7]]. In recent years, efferocytosis-enhancing therapies have demonstrated promising therapeutic effects in various fields, including myocardial infarction, stem cell transplantation, and cancer treatment [[8], [9], [10]]. In atherosclerosis, failing to effectively and promptly clear apoptotic cells leads to secondary necrosis, exacerbating inflammation within plaques and increasing plaque instability [11]. Therefore, the timely and effective clearance of apoptotic cells can improve plaque inflammation and inhibit plaque progression.

Macrophages undergo three processes—“find me,” “eat me,” and “digestion and resolution”—to perform efferocytosis [12]. Through molecular signals, they clear and degrade apoptotic cells, thereby maintaining local tissue homeostasis and overall body equilibrium [13]. The CD47-SIRPα axis serves as a “do not eat me” signal that protects normal cells from being cleared by macrophages [14]. In atherosclerotic plaques, the abnormal elevation of CD47 hinders macrophage recognition of apoptotic cells [9]. Inhibitors of the CD47-SIRPα signaling axis, such as CD47 monoclonal antibodies and SHP1 inhibitors, can enhance macrophage efferocytosis in atherosclerosis, alleviating plaque inflammation and improving the condition of atherosclerosis [10,15]. Unlike the localized microenvironments in myocardial infarction or tumors, the high lipid and inflammatory environment within atherosclerotic plaques adversely affects macrophage metabolism [16]. During the efferocytosis process, macrophages adapt to the influx of large amounts of cholesterol by upregulating the expression of ABCA1 [17]. However, in the atherosclerotic environment, the abundance of lipid components, inflammatory cytokines, and the high levels of cholesterol and lipids within apoptotic cells promote the degradation of ABCA1 in macrophages, reducing cholesterol efflux [[18], [19], [20]]. This leads to macrophage foam cell formation and accelerates the progression of atherosclerosis [21,22]. Furthermore, the accumulation of cholesterol due to impaired cholesterol efflux pathways activates the NLRP3 inflammasome within macrophages. It alters the lipid composition of the macrophage membrane, affecting phagocytic receptors' functionality and resulting in efferocytosis defects [23]. This indicates that the inability to effectively and promptly expel excessive intracellular cholesterol is detrimental to the efferocytosis function of macrophages [19,24]. Therefore, appropriate regulation of lipid metabolism is essential for macrophages to maintain their efferocytosis capacity and promote the resolution of inflammation.

Biomimetic nanoparticles represent a promising platform for targeted therapy due to their unique properties [25]. Natural cell membranes confer a range of essential functions, such as “self” markers for immune recognition, biological targeting capabilities, and compatibility with the immune system [26]. In addition to acting as drug carriers, biomimetic nanoparticles enveloped in immune-cell membranes retain critical surface proteins that confer them with immune-cell-like homing abilities, allowing for selective accumulation at sites of inflammation [27]. These native membrane proteins are crucial for enhancing the therapeutic targeting and overall efficacy of the nanoparticles [8].

This study developed a combination therapy to promote macrophage phagocytosis and clearance of apoptotic cells, while simultaneously reducing the lipid burden in macrophages to enhance efferocytosis further, focusing on the entire efferocytosis process. Macrophage membranes were isolated *in vitro*, and lipid-soluble retinoic acid was loaded into liposomes. The macrophage membranes were then fused with the retinoic acid-loaded liposomes through extrusion, creating retinoic acid-loaded macrophage membrane-biomimetic liposome (R@MLP). On the one hand, the adhesion molecules carried by the macrophage membrane components in R@MLP can target the surface of apoptotic cells within the plaques. At the same time, SIRPα can block the abnormally elevated CD47 on the surface of apoptotic cells. This action hinders the recognition of CD47 by macrophage SIRPα, thereby promoting the phagocytic clearance of apoptotic cells by macrophages. On the other hand, the retinoic acid carried by R@MLP enters phagocytic macrophages and apoptotic cells, further activating LXR, which promotes the expression of ABCA1 and ABCG1, ultimately facilitating lipid efflux. The synergistic effects of these two mechanisms collectively enhance the efferocytosis of macrophages throughout the entire process within the plaques, reduce plaque inflammation, and improve the condition of atherosclerosis.

## 2 Method

### 2.1 Animals and cell lines

Male ApoE^−/−^ mice were purchased from Shanghai Jiesijie Laboratory Animal Co., Ltd (6 weeks old). Animal experiments were approved by the Ethics Committee of Zhongshan Hospital, Shanghai, China. Raw264.7 and HUVEC cell lines were purchased from the Institute of Biochemistry and Cell Biology, Chinese Academy of Sciences (Shanghai, China). Before the experiments, all animals were housed under standard conditions for at least three days to acclimatize.

### 2.2 Isolation and verification of macrophage membrane

Macrophage membranes (M) were acquired using a previously reported method with a minor modification [28,29]. Specifically, RAW264.7 macrophage cells (cell number ≈ 1 × 10^8^) were isolated from a culture dish with 0.25 % Trypsin–EDTA, washed with PBS 3 times (500×*g* for 10 min each time), and dispersed in PBS. Next, a hypotonic lysing buffer containing one mmol/l NaHCO [3], 0.2 mmol/l EDTA, and 1 mmol/l PMSF were added to disperse cells and incubated at 4 °C overnight. The cell suspension was then put into a non-contact automatic ultrasonic disruptor and destroyed after 60 cycles. The resulting suspension was centrifuged at 3200×*g* for 5 min at 4 °C to remove large debris. The collected supernatant was centrifuged at 20,000×*g* for 25 min to remove the pellet. Finally, the supernatant was centrifuged at 100,000×*g* for 35 min to collect the cell membranes in the bottom, which were dispersed in PBS (pH = 7.4). Macrophage membrane vesicles were formed by passing the extracted macrophage membranes 15–20 times through 400 nm and 200 nm polycarbonate membranes with an Avestin Mini-extruder. The harvested macrophage membrane vesicles were stored in PBS at 4 °C for later use.

Macrophage membranes devoid of SIRPα (MKD) were generated through a two-step process. SIRPα was knocked down in the RAW264.7 cell line using SIRPα-specific shRNA to construct a stable knockdown cell line. Subsequently, these genetically modified cells were subjected to the aforementioned membrane extraction protocol.

### 2.3 Synthesis of LP and R@LP

According to the previously published article, conventional liposomes were prepared using the thin-film hydration and extrusion method with minor modifications [8]. Briefly, Conventional liposome (LP) was synthesized by 3.6 mg 1,2-Dimyristoyl-sn-glycero-3-Phosphocholine (DMPC), 0.4 mg 1,2-distearoyl-sn-glycero-3-phosphoethanolamineN-[methoxy(polyethyleneglycol)−2000] (DSPE-PEG2000) through film hydration. For 9-cis-retinoic acid (RA) loading, 4 mg 9-cis-retinoic acid solution was added to 3.6 mg 1,2-Dimyristoyl-sn-glycero-3-Phosphocholine (DMPC), 0.4 mg 1,2-distearoyl-sn-glycero-3-phosphoethanolamineN-[methoxy(polyethyleneglycol)−2000] (DSPE-PEG2000) through film hydration to form R@LP nanoparticles. For nanoparticle tracing, 1,1′dioctadecyl-3,3,3′,3′-tetramethylindotricarbocyanine perchlorate (DiD) was added (1:1000). The solution was extruded through 0.4, 0.2, and 0.1 μm polycarbonate membranes sequentially (Nuclepore Track-Etched Membranes, Whatman, UK), using a LiposoFast extruder apparatus (Avestin, Canada) to get final LP.

### 2.4 Preparation of MLPKD, R@MLPKD, MLP and R@MLP

A mechanical co-extrusion method was used to fuse the macrophage membrane and the LP or R@LP nanoparticles to obtain M or MKD biomimetic nanoparticles. Firstly, the collected M or MKD vesicles were mixed with nanoparticle cores with a membrane protein-to-polymer weight ratio of 1:1, and the mixture was sonicated with a bath sonicator at a frequency of 42 kHz and a power of 100 W for 2 min. Then, the mixture was extruded 15–20 times through a mini-extruder to obtain MLPKD, R@MLPKD, MLP, and R@MLP nanoparticles. Finally, the solution was centrifuged at 10,000×*g* for 30 min to remove the uncoated membrane. Several different types of prepared nanoparticles were stored at 4 °C for subsequent experiments.

### 2.5 Construction of AS in ApoE^−/−^ mice

Six-week-old ApoE^−/−^ mice were given a high-fat diet (HFD) containing 20 % fat and 1.25 % cholesterol to induce atherosclerosis. All animal experiments were approved by the Institutional Animal Care and Use Committee (IACUC) of Zhongshan Hospital, Fudan University (Approval No. 2023-283; Date: 11/2023) and adhered to the ARRIVE guidelines for reporting *in vivo* research.

### 2.6 Bone marrow-derived macrophages(BMDMs)

BMDMs were acquired using a previously reported method with a minor modification [30,31]. Cells were obtained by flushing the femur and tibia from C57BL/6 mice (6–8 weeks), and the isolated cells were cultured in culture medium (Dulbecco's modified Eagle's medium (DMEM) supplemented with 10 % fetal bovine serum (FBS), 1 % penicillin and streptomycin), and 10 ng/ml Macrophage colony-stimulating factor(M-CSF). The culture medium was changed every 3 days, and cells were collected on day 7.

### 2.7 Construction of foam cell-derived apoptotic cells (FCDACs)

FCDACs were acquired using a previously reported method with a minor modification [32]. Foam cells were generated by incubating RAW264.7 cells with 100 μg/ml oxidized low-density lipoprotein (ox-LDL) and 10 μg/ml of the acyl-coenzyme A: cholesterol acyltransferase inhibitor 58035 (Sigma-Aldrich) for 20 h. Subsequently, the foam cells were treated with 2.5 μM staurosporine for 3 h to induce apoptosis. The cells were centrifuged at 3000 rpm for 5 min to remove excess low-density lipoprotein and staurosporine, preparing them for subsequent experiments.

### 2.8 Preparation of nanoparticles-decorated FCDACs(NPs-decorated FCDACs)

FCDACs were prepared by staining with DiD dye and subjected to a 30-min pretreatment with either phosphate-buffered saline (PBS), MLPKD, R@MLPKD, MLP, or R@MLP. After incubation, the cells were collected by centrifugation at 1000 rpm for 5 min. The supernatant was discarded, and the NPs-decorated FCDACs were resuspended in fresh complete medium for subsequent use.

### 2.9 *In vitro* efferocytosis assay

In brief, BMDMs were seeded in 35 mm glass-bottom culture dishes at a density of 3 × 10^5 cells per well. NP-decorated FCDACs were co-incubated with BMDMs in fresh complete medium for 2 h at a 5:1 (FCDACs: BMDMs) cell ratio. Following the co-incubation period, the culture medium was aspirated, and the cells were fixed with paraformaldehyde. Macrophage cell membranes were then labeled with Texas-Red-labeled wheat germ agglutinin (WGA). Efferocytosis activity was observed and quantified through confocal microscopy and flow cytometry, enabling a comprehensive high-resolution assessment of phagocytic efficiency under various treatment conditions.

### 2.10 *In vitro* cholesterol efflux assay

NP-decorated FCDACs were co-incubated with BMDMs in fresh complete medium for 2 h at a 5:1 (FCDACs: BMDMs) cell ratio. Subsequently, the medium was removed, and the cells were washed three times with PBS to eliminate unphagocytosed apoptotic cells. BMDMs were incubated with 10 μM BODIPY-cholesterol for 2 h, after which the labeling solution was replaced with fresh complete medium.

To quantify the cholesterol efflux rate, time-lapse confocal microscopy was employed to acquire images of the same microscopic field over a duration of 60 min. The total mean fluorescence intensity for each cell was determined by normalizing the fluorescence intensity to the corresponding cell area. The difference in mean fluorescence intensity between the time points of 0 and 60 min was subsequently divided by the 60-min interval to calculate the efflux rate for each cell.

For efflux-ratio analysis, cells were incubated for 4 h in fresh complete medium, and the culture supernatant and cell pellet were collected separately. Their fluorescence was then measured using a microplate reader. The efflux ratio was calculated as fluorescence_supernatant/(fluorescence_supernatant + fluorescence_cells).

Oil Red O staining assessed intracellular lipid droplet content and reflected cholesterol efflux levels in RAW 264.7.

### 2.11 In vitro inflammatory factor RNA expression level detection

After co-incubating with NPs-decorated FCDACs for 2 h, the RAW264.7 cells were then replaced with fresh complete medium for 24 h. RNA isolation was performed with the Super FastPure Cell RNA Isolation Kit (RC102-01, Vazyme Biotech Co., Ltd). qRT-PCR was performed to quantify mRNA expression of actin (internal control), pro-inflammatory cytokines (IL-1β, IL-6, TNF-α), and immunoregulatory markers (IL-10, TGF-β, Arg1).

### 2.12 *In vivo* targeting of atherosclerotic plaque

Eight-week-old female ApoE^−/−^ mice fed a high-fat diet for 4 weeks were administered with PBS, MLPKD, and MLP via the tail vein, respectively. Two hours later, the mice were euthanized and perfused with PBS to clear any remaining blood and unbound dyes. Using a chemiluminescence/fluorescence image analysis system, the aortas were isolated for imaging and quantitative analysis.

### 2.13 *In vivo* treatment of AS in ApoE^−/−^ mice

ApoE^−/−^ mice were initially fed a high-fat diet for 4 weeks and then randomly divided into five groups, receiving injections of PBS, MLPKD, R@MLPKD, MLP, or R@MLP every 7 days for a total of 8 weeks. The ApoE^−/−^ mice were euthanized at the end of the treatment period. Oil Red O staining was performed on the aorta from the heart to the iliac bifurcation to determine the extent of atherosclerosis to assess the plaque area. For histological examination, frozen aortic root sections from mice given various treatments were collected and then subjected to hematoxylin-eosin (H&E), Masson's trichrome, Oil Red O, and terminal deoxynucleotidyl transferase dUTP nick end labeling (TUNEL) staining.

### 2.14 In vivo efferocytosis assay

We defined apoptotic cell debris that macrophages had phagocytosed as TUNEL-positive signals that did not colocalize with DAPI. Among these, TUNEL signals that colocalized with the macrophage marker F4/80 were counted as macrophage-associated, whereas those without F4/80 colocalization were considered free TUNEL signals, enabling an *in vivo* assessment of efferocytosis efficiency. We further quantified TUNEL-positive signals that colocalized with DAPI and the macrophage marker F4/80, defining these triple-positive cells as apoptotic macrophages and using their frequency to indicate plaque-level apoptosis.

### 2.15 Statistical analysis

The statistical analysis relied on Student's t-test, one-way and two-way analysis of variance (ANOVA) methods using GraphPad Prism (v8.0.1). p < 0.05 was considered statistically significant. All data were presented as the mean value ± s.d.

## 3 Result

### 3.1 Preparation, characterization, and CD47 blocking efficiency of R@MLP

**Fig. 1:**
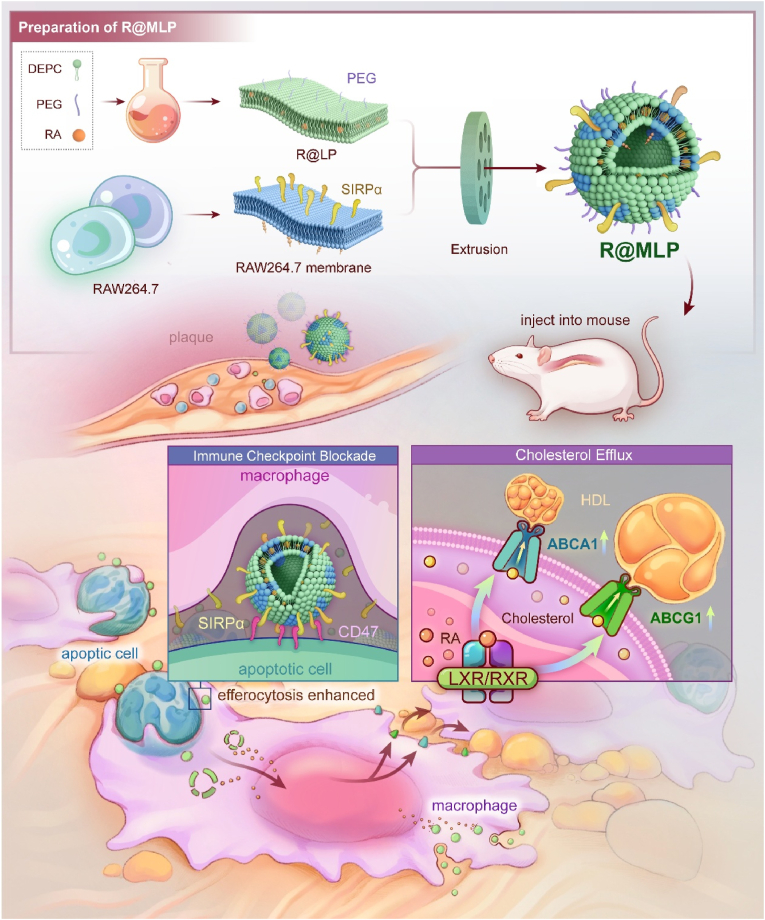
Schematic representation of R@MLP fabrication and its dual-action mechanism in pro-efferocytosis therapy. This novel nanoparticle system is designed to synergistically inhibit immune checkpoints and promote cholesterol efflux, functioning analogously to digestive aids in its approach to ameliorating atherosclerosis. The R@MLP construct combines checkpoint inhibition with enhanced lipid metabolism to effectively clear apoptotic cells and reduce plaque formation.

As illustrated in Fig. 1, liposomes were synthesized using the thin-film hydration method. The initial organic phase combined retinoic acid, DMPC, and PEG. Hydration was performed after organic solvent removal via rotary evaporation under negative pressure. The lipophilic retinoic acid was encapsulated within the DMPC lipid bilayer spheres, forming retinoic acid-loaded liposomes (R@LP). The acquisition of macrophage membranes was performed as described above. Following the isolation of the macrophage membranes, they were mixed with R@LP. The mixture underwent sonication and extrusion to produce retinoic acid-loaded macrophage membrane-biomimetic liposomes (R@MLP). Additionally, unloaded macrophage membrane-biomimetic liposomes (MLP) were constructed as a CD47 blockade control. Furthermore, SIRPα-knockdown Raw264.7 cell lines generated macrophage membranes lacking CD47 blocking functionality. These were used to build SIRPα-knockdown macrophage membrane-biomimetic liposomes (MLPKD) and retinoic acid-loaded SIRPα-knockdown macrophage membrane-biomimetic liposomes (R@MLPKD), serving as non-functional and lipid efflux-promoting controls, respectively.

**Fig. 2:**
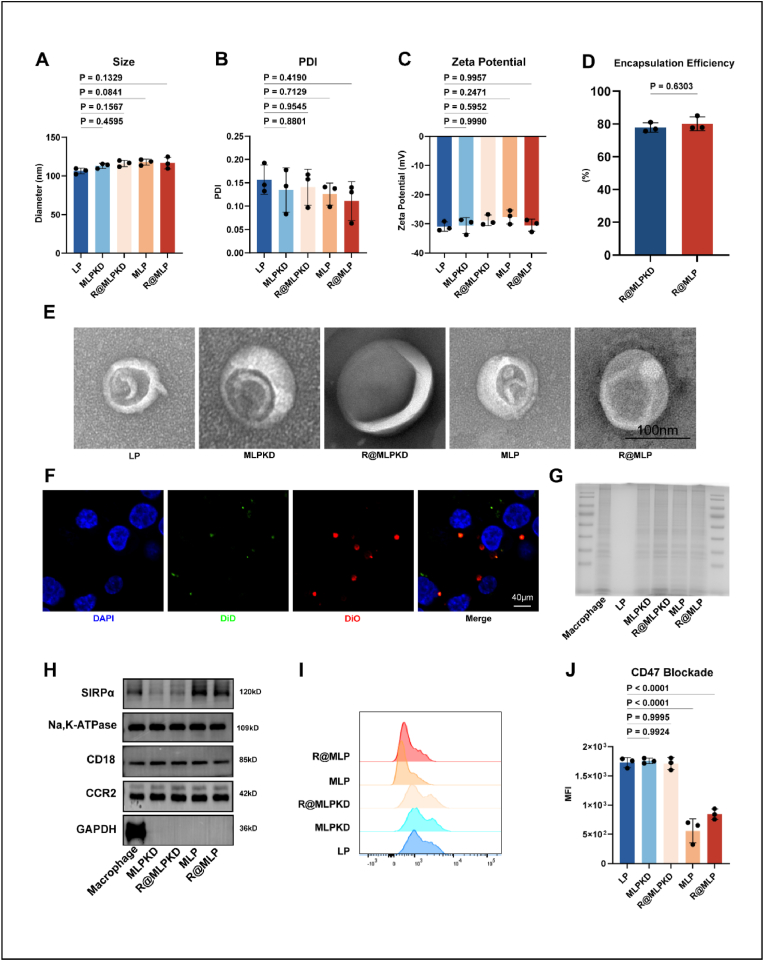
Preparation, characterization, and CD47 blocking efficiency of R@MLP (A) Diameter, (B) PDI, and (C) Zeta potential of LP, MLPKD, R@MLPKD, MLP, and R@MLP. Statistical analysis was calculated using the one-way ANOVA and Tukey's tests. (D) Encapsulation efficiencies of retinoic acid in R@MLPKD and R@MLP. Statistical analysis was calculated using the two-sided Student's t-test (n = 3). (E) TEM images of LP, MLPKD, R@MLPKD, MLP, and R@MLP. (F) CLSM images of extruded M(red) and LP (green) after incubation with Raw264.7 cells for 20 min. (G) Coomassie blue staining of macrophage membrane, LP, MLPKD, R@MLPKD, MLP, and R@MLP. (H) Western blotting for 3 key proteins associated with chemotaxis and CD47 blockade in LP, MLPKD, R@MLPKD, MLP, and R@MLP. (I) CD47 blockade efficiency of LP, MLPKD, R@MLPKD, MLP, and R@MLP, with (J) quantitative assays. Statistical analysis was calculated using the one-way ANOVA and Tukey's tests.

Dynamic Light Scattering (DLS) was employed to measure the particle size, zeta potential, and polydispersity index (PDI) of LP, MLPKD, R@MLPKD, MLP, and R@MLP. No significant differences in particle size were observed among the groups, with mean diameters of 103.04 ± 3.88, 113.06 ± 3.23, 116.20 ± 3.97, 117.79 ± 3.81, and 116.63 ± 7.60 nm, respectively (Fig. 2A). Similarly, zeta potentials showed no significant variations: 30.95 ± 1.59, −30.62 ± 2.74, −28.85 ± 1.84, −27.65 ± 2.37, and −30.47 ± 2.08 mV, respectively (Fig. 2C). All groups demonstrated good dispersity (Fig. 2B). The encapsulation efficiencies of retinoic acid in R@MLPKD and R@MLP were determined to be 77.84 ± 2.85 % and 80.11 ± 4.21 %, respectively (Fig. 2D). The drug loading of retinoic acid in R@MLPKD and R@MLP was determined to be 1.282 ± 0.28 %, 1.16 ± 0.22 %, respectively (Fig. S1D). Transmission electron microscopy (TEM) revealed R@MLP as spherical, bilayered structures, showing no significant morphological differences among LP, MLPKD, and MLP (Fig. 2E). Stability was assessed by storing the particles in PBS for 7 and 28 days. Particle size, zeta potentials, PDI, and TEM measurements on day 7 and day 28 showed no significant changes compared to day 0, indicating excellent stability of R@MLP (Fig. S1A–D). To verify the complete fusion of cell membranes with liposomes, the cell membranes were labeled with DiO, while the liposomes were labeled with DiD. Following co-extrusion, R@MLP was incubated with Raw264.7 cells for 20 min. Confocal Laser Scanning Microscope (CLSM) confirmed the co-localization of both components post-extrusion (Fig. 2F). Coomassie blue staining revealed similar protein compositions among the four nanoparticle types (Fig. 2G). Western blot analysis revealed significant differences in SIRPα content among liposomes constructed with various macrophage membranes, with MLPKD and R@MLPKD exhibiting markedly reduced levels of SIRPα (Fig. 2H an S1E). These results confirm the successful construction of four distinct nanoparticle types for subsequent experiments.

To evaluate the liposomes' CD47-blocking capacity, apoptotic cells were co-incubated with different liposomes for 2 h. After centrifugation to remove unbound nanoparticles, the CD47-blocked apoptotic cells were obtained. Flow cytometry analysis of FITC-labeled CD47 antibody-stained cells revealed a notable leftward shift in the MFI peak, indicating effective CD47 blockade (Fig. 2I and J). The incorporation of retinoic acid did not affect the liposomes' CD47-blocking efficiency.

Retinoic acid-loaded macrophage membrane-biomimetic liposomes (R@MLP) were successfully prepared.

### 3.2 Targeting ability of R@MLP *in vitro* and *in vivo*

**Fig. 3:**
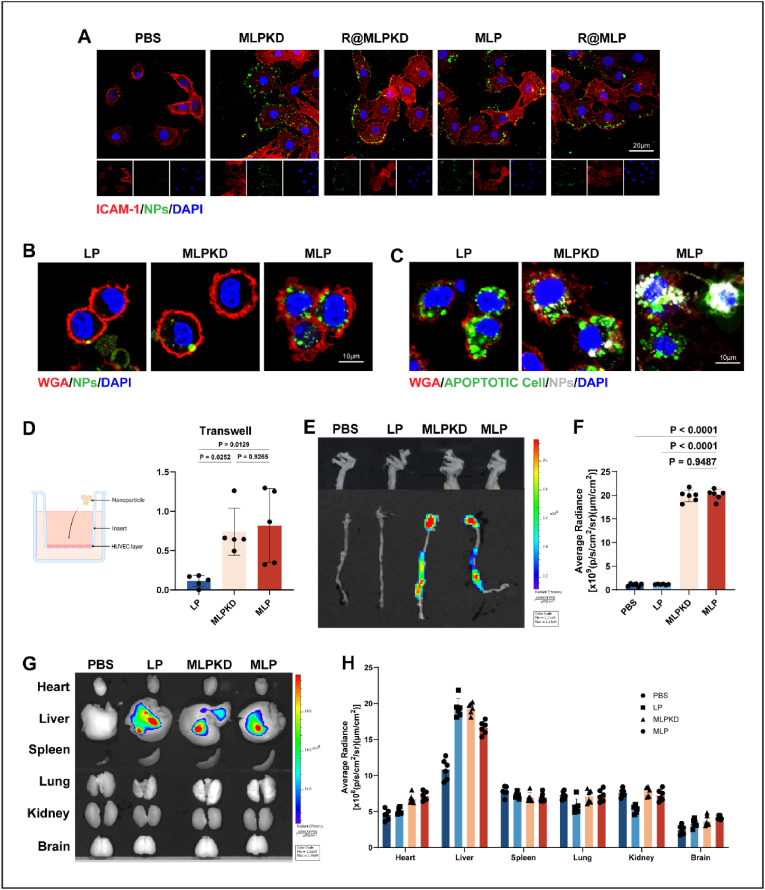
Targeting ability of R@MLP *in vitro* and *in vivo* (A) Representative immunostaining images of binding of LP, MLPKD, R@MLPKD, MLP, and R@MLP to TNFα pretreated HUVECs were imaged after staining for ICAM-1. (B) Representative immunostaining images of FCDACs incubated with DiD-labeled LP, MLPKD, and MLP for 2 h. (C) Representative immunostaining images of NPs-decorated FCDACs phagocytosed by BMDMs. (D) Schematic diagram of the transwell permeability model. Fluorescence intensity in the different compartments of the transwell system was recorded after treatment with LP, MLPKD, and MLP(n = 5). Statistical analysis was calculated using the one-way ANOVA and Tukey's tests. (E) Ex vivo imaging of aortas from ApoE^−/−^ mice after incubating with different nanoparticles *in vitro* and (F) corresponding semi-quantitative results of optical imaging of aortas (n = 6). Statistical analysis was calculated using the one-way ANOVA and Tukey's tests. (G) Representative images of significant organs by ex vivo IVIS in PBS, MLPKD, and MLP group 2 h after injection. And (I) corresponding semi-quantitative optical imaging results of heart, liver, spleen, lung, kidney, and brain(n = 6). Statistical analysis was calculated using the one-way ANOVA and Tukey's tests.

We next evaluated whether R@MLP could improve the targeting ability of liposomes. Our previous studies demonstrated that cell membrane-mimicking nanoparticles mainly rely on interacting with surface adhesion molecules (CCR2, CD18) and ICAM-1 to display chemotactic properties toward inflammatory sites [33,34]. HUVECs were treated with TNFα for 6 h before adding different nanoparticles. We observed that LP (unmodified liposomes) could not interact with ICAM-1 on the surface of inflammation-induced endothelial cells, thus failing to adhere to the endothelial cell surface. In contrast, R@MLP showed significant co-localization (Fig. 3A). This suggests that membrane-biomimetic liposomes can effectively target inflammatory sites.

To confirm whether nanoparticles can adhere to FCDACs, they were co-incubated with FCDACs for 2 h. Confocal images show that MLP accumulated on the membrane of FCDACs more than MLPKD and LP (Fig. 3B), indicating that MLP can adhere to FCDACs with high affinity. We further co-incubated the NPs-decorated FCDACs with BMDM for 2 h to verify whether macrophages can uptake both FCDACs and nanoparticles. Confocal images indicate increased colocalization of FCDACs and nanoparticles within a single macrophage in the MLP group (Fig. 3C). Overall, this data confirms that MLP effectively adheres to HUVEC and can be taken up by macrophages.

Subsequently, we constructed a Transwell model to test R@MLP's ability to traverse endothelial cells. HUVECs were cultured in 0.4 μm hanging cell culture inserts until reaching 100 % confluence. After treatment with TNFα for 6 h, DiD-labeled nanoparticles were added. We found that the fluorescence of membrane-biomimetic liposomes in the lower chamber was significantly higher than that of liposomes without membrane modification (Fig. 3D). These results suggest that R@MLP inherits the surface characteristics of macrophages, allowing it to accumulate in inflammatory areas, and intermolecular interactions enable it to cross endothelial cells and enter the interstitial space.

We then verified the ability of R@MLP to target atherosclerotic plaques *in vivo*. ApoE^−/−^ mice were fed a Western diet for 4 weeks to establish an atherosclerosis model for further experiments. After administering the formulations via tail vein injection, mice were sacrificed 2 h post-administration, and various organs and the aorta were collected for ex vivo fluorescence imaging. The results showed that DiD-labeled R@MLP and R@MLPKD mainly accumulated in atherosclerotic plaques (Fig. 3E and F) compared to empty liposomes. Although SIRPα was knocked out, the targeting ability of R@MLP and R@MLPKD primarily relies on CD18 and CCR2 expressed on the macrophage membrane. As a result, no significant differences in fluorescence intensity were observed between the two groups in in vivo imaging. Still, no significant uptake was observed in the heart, liver, spleen, lung, kidney, brain, and other tissues (Fig. 3G and H). These results indicate that R@MLP is a formulation capable of effectively delivering its payload to plaque areas.

### 3.3 Promoting efferocytosis and cholesterol efflux *in vitro*

**Fig. 4:**
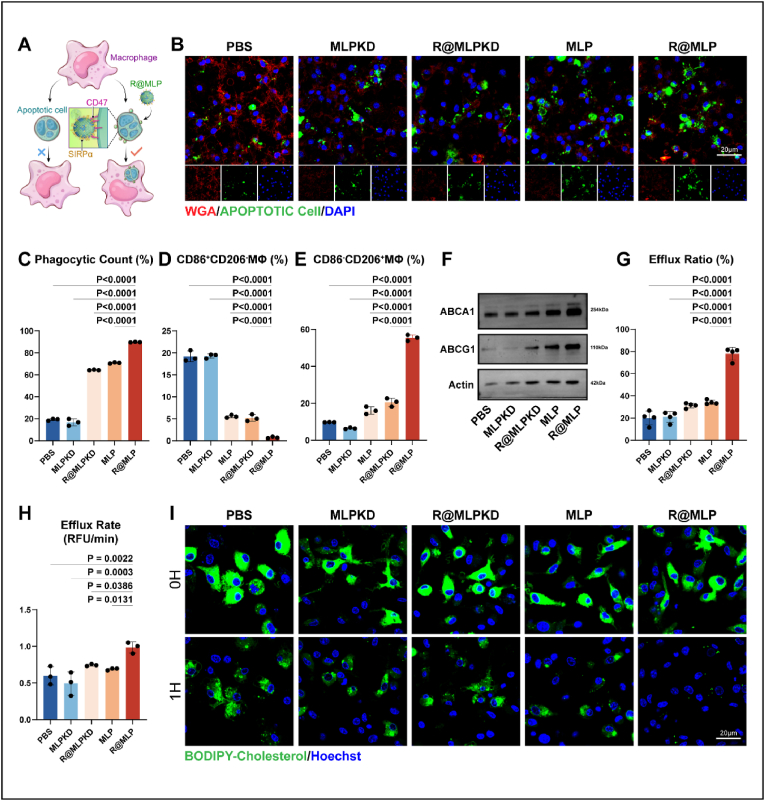
Promoting efferocytosis and cholesterol efflux *in vitro* (A) Schematic diagram of the relationship between CD47-SIRPa axis inhibition and efferocytosis enhancement. (B) CLSM images of BMDMs that were incubated with DiD-labeled NPs-decorated FCDACs for 2 h, then stained with Texas red-labeled WGA to show cell membranes (red) (C) Percent efferocytosis was quantified as the number of macrophages with engulfed apoptotic cells as a percentage of total macrophages(n = 3). Statistical analysis was calculated using the one-way ANOVA and Tukey's tests. (D) The percentage statistics of CD86^+^ and CD206^-^ positive macrophages(n = 3). Statistical analysis was calculated using the one-way ANOVA and Tukey's tests. (E) The percentage statistics of CD86^−^ and CD206^+^ positive macrophages(n = 3). Statistical analysis was calculated using the one-way ANOVA and Tukey's tests. (F) Western blotting for 2 key proteins associated with cholesterol efflux in BMDMs after treatment with different nanoparticles and FCDACs. (G) Cholesterol efflux ratio of BMDMs(n = 3). Statistical analysis was calculated using the one-way ANOVA and Tukey's tests. (H) Cholesterol efflux rate of BMDMs(n = 3). Statistical analysis was calculated using the one-way ANOVA and Tukey's tests. (I) A time-lapse CLSM image of BMDMs treated with BODIPY-labeled cholesterol for 2 h, then replaced with serum-free medium and scanned for 1 h.

We first validated the successful preparation of the FCDACs model. Raw264.7 cells were co-incubated with ox-LDL for 24 h, followed by staurosporine treatment for 2 h to induce foam cell apoptosis. Western blot analysis of caspase-3 confirmed the establishment of the foam cell apoptosis model (Fig. S2A–C). To demonstrate the efferocytosis-promoting capability of R@MLP, apoptotic cells were pretreated with R@MLP for 2 h before being co-incubated with BMDM. Immunofluorescence results revealed that R@MLP significantly enhanced the phagocytic clearance of apoptotic cells by macrophages (Fig. 4B). These findings were further corroborated by flow cytometry analysis, which confirmed the enhanced efferocytosis-promoting ability of R@MLP (Fig. 4C and Fig. S3).

Next, the impact of R@MLP on the polarization state of macrophages was examined. Flow cytometry results indicated a significant increase in the proportion of CD206-positive macrophages in the R@MLP group, reflecting a shift towards an anti-inflammatory phenotype after enhanced efferocytosis (Fig. 4D and E, and S4A). qPCR results confirmed increased RNA expression of anti-inflammatory factors and decreased expression of pro-inflammatory factors in macrophages (Fig. S5A and S5B).

Subsequently, we investigated whether the retinoic acid carried by R@MLP could promote cholesterol efflux from macrophages. Western blot analysis revealed that R@MLP significantly increased the expression of ABCA1 and ABCG1 in macrophages (Fig. 4F and Fig. S5C). To further confirm that cholesterol was indeed being expelled from the macrophages, we labeled the treated BMDMs with BODIPY-labeled cholesterol. BMDMs were continuously cultured for 4 h in a medium without BODIPY-labeled cholesterol, then, supernatants and cells were collected to quantify the total cholesterol efflux ratio. In the PBS group and the MLPKD group, 20.07 % ± 6.34 % and 21.04 % ± 4.82 % cholesterol efflux from BMDM, respectively. R@MLPKD group and MLP group increase efflux ratio to 31.30 % ± 2.17 and 34.25 % ± 1.94, respectively. In the R@MLP group, 77.98 % ± 5.79 % cholesterol was effluxed from the cell, much higher than in other groups(Fig. 4G). We also continuously imaged the same field of view after removing BODIPY-labeled cholesterol medium, and the cholesterol efflux rate was quantified. The immunofluorescence results indicated that R@MLP significantly accelerates the efflux rate (Fig. 4H and I). Additionally, Oil Red O staining was performed, which demonstrated a significant reduction in lipid droplets within the macrophages (Fig. S6A and S6B). Therefore, R@MLP can promote the phagocytic clearance of apoptotic cells by macrophages while simultaneously reducing their lipid burden.

We also observed LXRα, a transcription factor activated by retinoic acid that then upregulates ABCA1 and ABCG1 expression, translocating into the nucleus. The confocal image shows that the nuclear/cytoplasmic ratio of LXRα significantly increased in the R@MLP group (Fig. S7A and B).

These results demonstrate that the SIRPα on R@MLP's surface effectively blocked CD47 on apoptotic cells, promoting the phagocytic process. Furthermore, as macrophages phagocytose apoptotic cells along with R@MLP, the increased expression of ABCA1 and ABCG1 facilitates the efflux of excess cholesterol from the macrophages. By combining these two effects, R@MLP ultimately enhanced macrophage efferocytosis and promoted the transition of macrophages toward an anti-inflammatory phenotype.

### 3.4 Anti-atherosclerosis efficacy by R@MLP

**Fig. 5:**
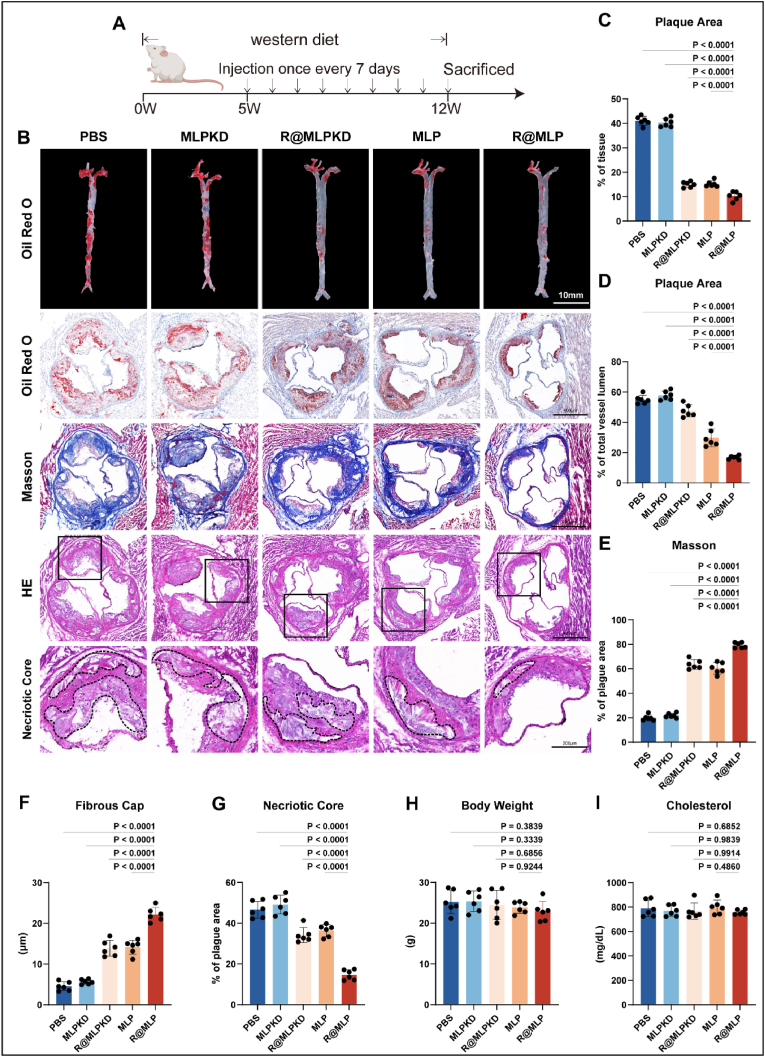
Anti-atherosclerosis efficacy by R@MLP (A) The *in vivo* experimental design timeline. (B) ORO-stained aorta tissues collected from atherosclerotic mice after different treatments. Representative photographs of aorta root sections stained by ORO, H&E, Masson's trichrome, and an enlarged image of the necrototic core. The black dotted lines indicate the necrotic core. (C) Quantitative analysis on plaque area of aorta tissues(n = 6). Statistical analysis was calculated using the one-way ANOVA and Tukey's tests. (D) Quantitative analysis on lumen area(n = 6). Statistical analysis was calculated using the one-way ANOVA and Tukey's tests. (E) Quantitative analysis on relative collagen content(n = 6). Statistical analysis was calculated using the one-way ANOVA and Tukey's tests. (F) Minimal cap thickness was measured at the thinnest region of the fibrotic cap surrounding the necrotic core in Masson's trichrome(n = 6). Statistical analysis was calculated using the one-way ANOVA and Tukey's tests. (G) Quantitative analysis of the necrotic core relative to plaque area by ImageJ software (n = 6). Statistical analysis was calculated using the one-way ANOVA and Tukey's tests. (H) Body weight(n = 6). Statistical analysis was calculated using the one-way ANOVA and Tukey's tests. (I) Cholesterol levels(n = 6). Statistical analysis was calculated using the one-way ANOVA and Tukey's tests.

To validate the therapeutic efficacy of R@MLP in atherosclerosis, we designed an experiment (Fig. 5A). Four-week-old ApoE^−/−^ mice were fed a high-fat diet throughout the study. From the fifth week of high-fat feeding, intravenous injections were administered via the tail vein every 7 days for 8 injections. Mice were euthanized 7 days after the final injection. The PBS-treated group exhibited significant plaque formation, covering 55.02 ± 2.89 % of the entire aorta (Fig. 5B and C). The MLPKD group, serving as a negative control, exhibited a plaque area similar to that of the PBS group (57.75 ± 2.99 %). The R@MLP and R@MLPKD groups demonstrated reduced plaque areas of 47.64 ± 4.057 % and 29.79 ± 5.78 %, respectively (Fig. 5B and C). Notably, R@MLP, through its synergistic effect of CD47 blockade and cholesterol efflux promotion, significantly reduced the plaque area to 16.81 ± 1.26 % (Fig. 5B and C). A similar trend was observed in plaque areas at the aortic valve (Fig. 5D). We also investigated the impact of R@MLP on plaque stability. Quantitative analysis revealed that plaques in the R@MLP group contained higher collagen content, indicating improved plaque stability (Fig. 5E). We further measured the minimum fibrous cap thickness, which was 4.53 ± 1.10, 5.65 ± 0.60, 13.88 ± 1.95, 14.17 ± 1.73, and 22.17 ± 1.73 μm, respectively. The R@MLP group displayed a significantly thicker minimum fibrous cap than all other groups(Fig. 5F). We also quantified the percentage of necrotic core area within the plaque, obtaining the following value: 46.68 ± 4.12 %, 49.15 ± 4.58 %, 34.18 ± 3.75 %, 36.36 ± 2.94 %, 14.68 ± 2.51 %. The R@MLP group exhibited a significantly lower necrotic core fraction(Fig. 5G).

It is worth noting that, as R@MLP primarily focuses on enhancing macrophage efferocytosis, no significant reductions in body weight or blood lipid levels were observed in any treatment group compared to the PBS group (Fig. 5H and I).

These *in vivo* results validate that R@MLP can effectively treat atherosclerosis in ApoE^−/−^ mice. The treatment significantly reduced atherosclerotic plaque area and improved plaque stability, demonstrating its therapeutic potential. However, it did not affect body weight or blood lipid levels. It was consistent with the *in vitro* findings emphasizing enhancing macrophage phagocytic function and improving cholesterol handling in macrophages rather than modulating systemic lipid metabolism like lipid-lowering therapies.

### 3.5 Promoting efferocytosis and cholesterol efflux *in vivo*

**Fig. 6:**
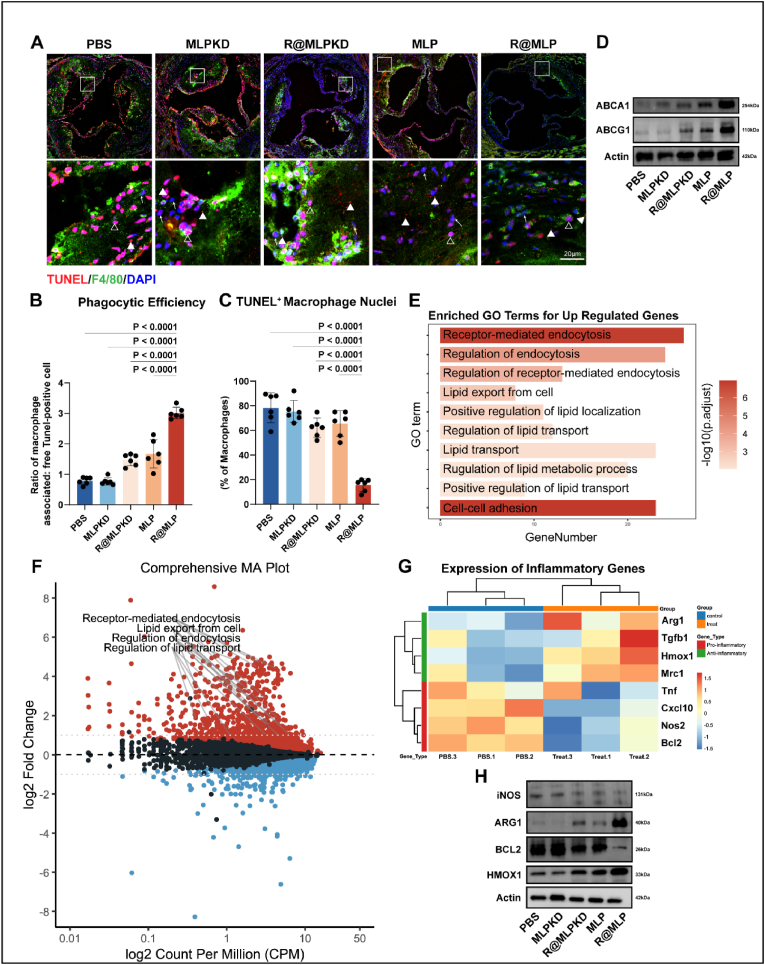
Promoting efferocytosis and cholesterol efflux *in vivo* (A) TdT-mediated dUTP Nick-End Labeling (TUNEL) staining of frozen sections of the aortic valve from ApoE^−/−^ mice and enlarged specific details. → represent live cells, △ depict apoptotic cells, and ▲ indicate TUNEL + cell debris, respectively. (B) Quantification of efferocytosis (macrophage-associated ACs:free ACs ratio) in aorta root sections(n = 6). Statistical analysis was calculated using the one-way ANOVA and Tukey's tests. (C) Quantification of apoptotic level (TUNEL + macrophage in all macrophages) in aorta root sections(n = 6). Statistical analysis was calculated using the one-way ANOVA and Tukey's tests. (D) Western blotting for 2 key proteins associated with cholesterol efflux in ApoE^−/−^ mice. (E) Gene Ontology (GO) enrichment analysis of differential expression genes (DEGs) between control and R@MLP-treated groups. (F) MA plot of DEGs between control and R@MLP-treated groups. (G) Heat map of expression of Inflammatory factors genes between control and R@MLP-treated groups. (H) Western blotting for 4 key proteins associated with the inflammation factor in in ApoE^−/−^ mice.

To validate whether R@MLP exhibits the exact therapeutic mechanism *in vivo* as observed *in vitro*, *in vivo* experiments were conducted to elucidate how R@MLP ameliorates atherosclerosis. Aortic valve sections from mice were subjected to TdT-mediated dUTP Nick-End Labeling (TUNEL) staining, and macrophages were labeled with F4/80. The *in vivo* apoptosis level and efferocytosis efficiency were quantified. The R@MLP group exhibited less macrophage apoptosis and a higher proportion of macrophage-associated apoptotic debris(Fig. 6A–C).

Furthermore, proteins extracted from whole aortas were analyzed via Western blot. Consistent with *in vitro* findings, the R@MLP group exhibited significantly elevated expression of ABCA1 and ABCG1 (Fig. 6D–S8A, and S8B). Additionally, RNA extracted from whole aortas was subjected to bulk RNA-seq analysis. Gene Ontology (GO) enrichment analysis revealed that transcriptional changes in the R@MLP group were predominantly enriched in pathways associated with endocytosis and cholesterol efflux (Fig. 6E and F). Further analysis was conducted on the changes in inflammatory factors following treatment with R@MLP. In the R@MLP treatment group, the expression of pro-inflammatory factors, such as TNFα, CXCL10, NOS2, and BCL2, decreased. In contrast, the expression of anti-inflammatory cytokines, including Arg1, TGFβ, HMOX1, and MRC1, was observed to increase(Fig. 6G). Western blot analysis also confirms these inflammatory factor' protein level change *in vivo*(Fig. 6H, and Fig. S8C–F). These findings are consistent with our *in vitro* results, providing evidence that R@MLP promotes the transition of macrophages to an anti-inflammatory phenotype, thereby alleviating plaque inflammation.

In conclusion, R@MLP demonstrated a therapeutic mechanism *in vivo* that parallels its *in vitro* effects, characterized by enhanced macrophage efferocytosis, upregulation of cholesterol efflux proteins, and modulation of gene expression pathways related to endocytosis and lipid efflux.

### 3.6 Biocompatibility of R@MLP

Finally, the biocompatibility of R@MLP was evaluated in healthy mice. 7 days post-injection, no significant morphological alterations were observed in major organs, including the liver, spleen, lungs, kidneys, and brain, compared to the PBS group (Fig. S9A). Compared with PBS injection, the concentration of IL-1β and TNF-α in plasma showed no significant elevation 7 days after R@MLP injection (Fig. S9B and S9C, Supporting Information). These findings demonstrate the safety profile of R@MLP in the murine model.

## 4 Discussion

Enhancing efferocytosis or cholesterol efflux—such as blocking the CD47-SIRPα axis with anti-CD47 antibodies or SHP1 inhibitors—attenuates atherosclerosis [9,10,15,35,36].

Our R@MLP meets this need by simultaneously blocking CD47-SIRPα and stimulating cholesterol efflux, thereby synergistically improving atherosclerosis. Efferocytosis-oriented therapies now include CD47/SHP1 antagonists, MerTK or LRP1 protectors, and nanoparticle systems that trigger apoptosis or deliver anti-inflammatory agents (e.g., effero-RLP-rosiglitazone) [9,10,[37], [38], [39]]. MLP alone raised phagocytosis; however, the resulting efflux still fell short of resolving foam-cell lipid. R@MLPKD also enhanced phagocytosis—likely via retinoic-acid activation of LXR/RAR pathways—whereas the control MLPKD behaved like PBS [40,41].

Previous research has demonstrated that SIRPα knockout in macrophages enhances cholesterol efflux without altering ABCA1 mRNA levels [42]. This explains why the MLP group, following the enhancement of phagocytosis, also exhibited increased expression of ABCA1 and ABCG1. Nevertheless, engulfing cholesterol-rich apoptotic foam cells overloads macrophages, so helping them process this lipid is an additional therapeutic need [43]. Isolated enhancement of efferocytosis or efflux (as in MLP or R@MLPKD) cannot offset the lipid gained from engulfing apoptotic foam cells, so both phagocytosis and cholesterol export remain inferior to the synergistic effect achieved by R@MLP [22]. Consequently, cholesterol accumulation blocks further efferocytosis, activates the NLRP3 inflammasome, seeds cholesterol crystals, and drives foam-cell apoptosis that accelerates atherosclerosis [44,45]. These insights, together with the β-CD LNP–based CAR-monocyte strategy [46], highlight the continuing need for interventions that eliminate intra- and extracellular cholesterol in atherosclerotic plaques.

Defective efferocytosis skews plaques toward pro-inflammatory macrophages, whereas efficient efferocytosis, together with robust cholesterol efflux, shifts them to an anti-inflammatory state and dampens plaque inflammation [45,[47], [48], [49]]. In our study, simultaneous CD47-SIRPα blockade and cholesterol-efflux stimulation reproduced this shift, allowing macrophages to clear apoptotic cells, unload excess lipids, and further suppress plaque inflammation.

R@MLP combines CD47-SIRPα blockade with retinoic-acid–driven cholesterol efflux, synergistically restoring macrophage function. Because macrophage efflux contributes only marginally to systemic lipid clearance [50], adjunct systemic lipid-lowering therapy is still required. Batch-to-batch variability in donor macrophage membrane composition may affect R@MLP's targeting and functional consistency, necessitating stringent source qualification and GMP-level quality control. Nonetheless, this macrophage-targeted, anti-inflammatory lipid-modulating strategy markedly eases plaque inflammation and lipid burden, supporting clinical translation [51]. We will next evaluate R@MLP in large-animal atherosclerosis models and explore catheter-based local delivery to maximize intraplaque exposure while minimizing systemic burden.

## 5 Conclusion

This study prepared macrophage membrane-biomimetic liposomes loaded with retinoic acid (R@MLP) to target and block the apoptotic cells with elevated CD47 levels in atherosclerotic plaques. This approach promotes macrophage phagocytosis while facilitating cholesterol efflux through the delivered retinoic acid, thereby improving the lipid burden in macrophages. The synergistic effects of these two mechanisms ultimately drive the transition of macrophages to an anti-inflammatory phenotype, reducing plaque inflammation. This anti-inflammatory and lipid-lowering combination therapy, which enhances phagocytosis and improves cholesterol metabolism, effectively ameliorates atherosclerosis and holds significant potential for clinical translation.

## CRediT authorship contribution statement

**Shiteng Cai:** Writing – original draft, Investigation, Conceptualization. **Jinfeng Gao:** Writing – original draft, Investigation, Conceptualization. **Xueyi Weng:** Methodology, Formal analysis. **Zhengmin Wang:** Visualization. **Danwen Zheng:** Validation, Data curation. **Qiaozi Wang:** Resources, Data curation. **Qiyu Li:** Visualization, Formal analysis. **Chengzhi Han:** Visualization. **Weiyan Li:** Validation. **Jing Chen:** Data curation. **Yuyuan Fu:** Validation. **Yiwen Tan:** Resources. **Bohan Wei:** Data curation. **Zhiqing Pang:** Writing – review & editing, Supervision, Project administration. **Zheyong Huang:** Writing – review & editing, Supervision, Funding acquisition. **Yanan Song:** Writing – review & editing, Conceptualization. **Junbo Ge:** Funding acquisition.

## Ethics approval and consent to participate

All animal experiments were approved by the Institutional Animal Care and Use Committee (IACUC) of Zhongshan Hospital, Fudan University (Approval No. 2023-283; Date: 11/2023) and adhered to the ARRIVE guidelines for reporting *in vivo* research.

## Declaration of competing interest

The authors declare that they have no known competing financial interests or personal relationships that could have appeared to influence the work reported in this paper.
