# Supplementary material for: Synergistic enhancement of efferocytosis and cholesterol efflux via macrophage biomimetic nanoparticle to attenuate atherosclerosis progression
Source: Bioact Mater. 2025 Sep 19;55:131–43. doi: 10.1016/j.bioactmat.2025.09.022 (PMC12481714; doi:10.1016/j.bioactmat.2025.09.022)
Supplement: Multimedia component 1 [file mmc1.docx]

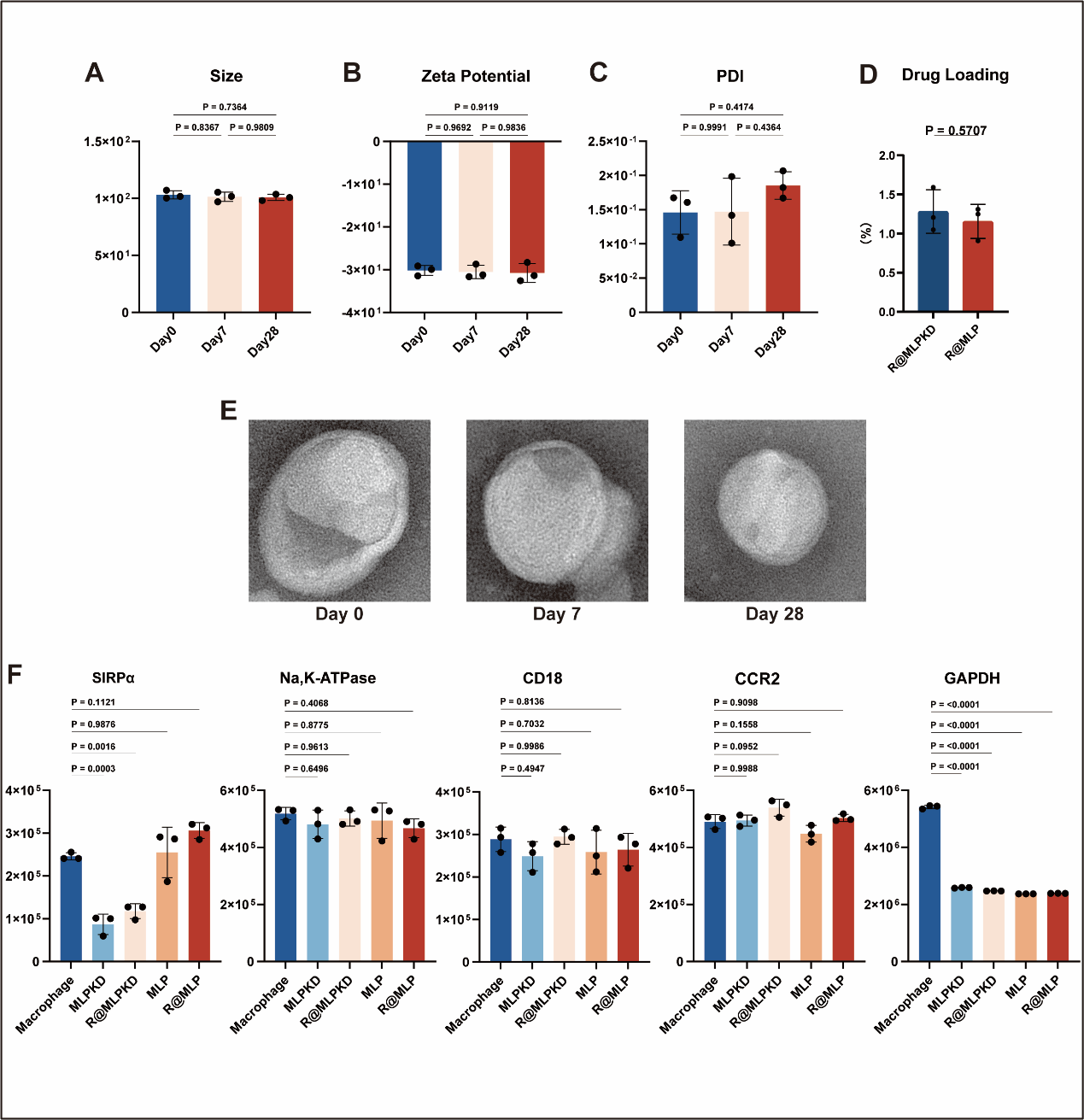


Figure.S1

(A) size, (B) zeta potential, and (C) polymer dispersity index (PDI)of R@MLP on day 0, 7, and 28 measured by dynamic light scattering(n=3). Statistical analysis was calculated using the one-way ANOVA and Tukey’s tests.

(D) Drug loading of R@MLPKD and R@MLP(n=3). Statistical analysis was calculated using the one-way ANOVA and Tukey’s tests.

(E) TEM images of R@MLP on days 0, 7, and 28.

(F) Quantification of Western blot analysis for 5 key proteins in macrophage biomimetic nanoparticles(n=3). Statistical analysis was calculated using the one-way ANOVA and Tukey’s tests.


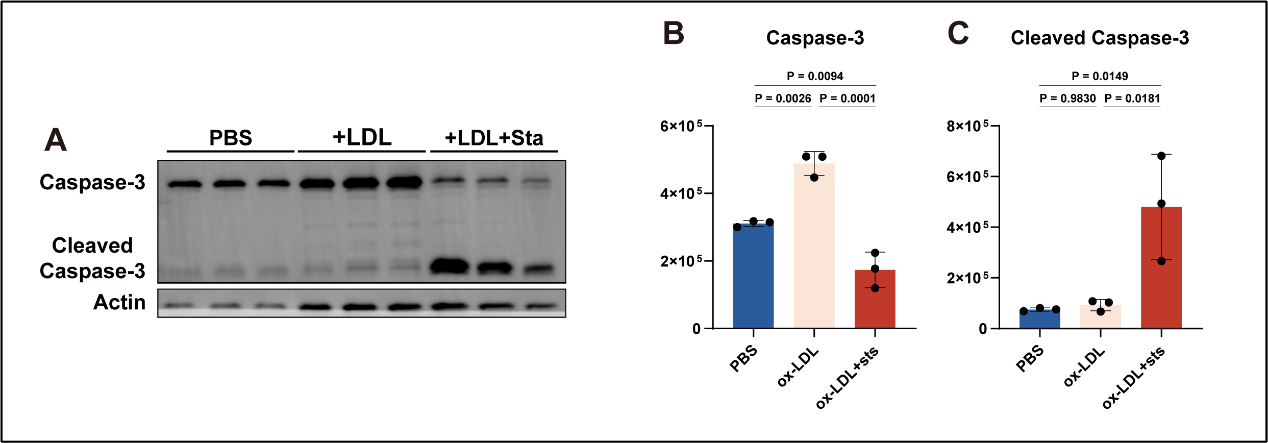


Figure.S2

(A) Western blotting for cleaved caspase-3 in different groups.

(B) and (C) Quantification of Western blot analysis for caspase-3 and cleaved caspase-3(n=3). Statistical analysis was calculated using the one-way ANOVA and Tukey’s tests.


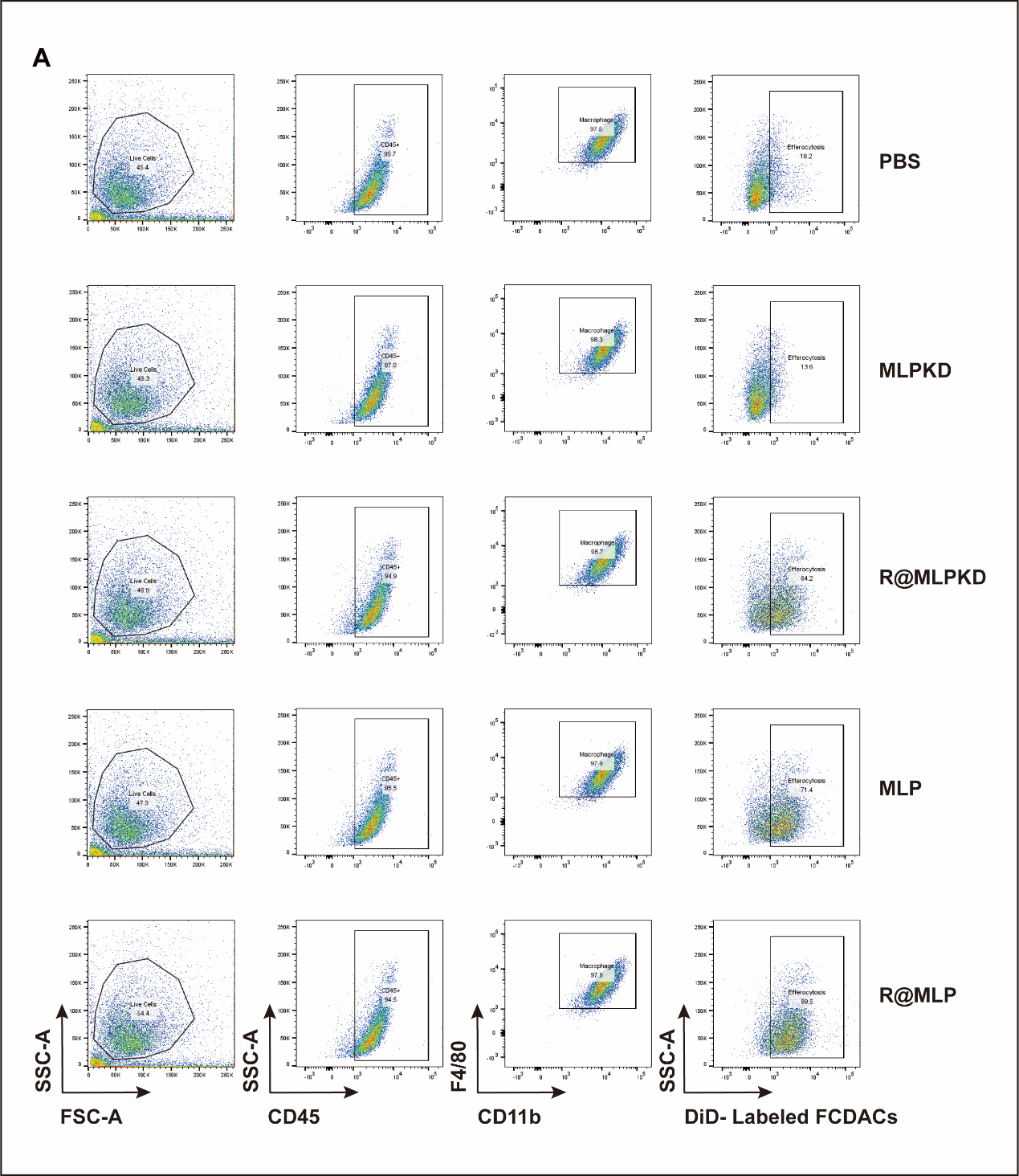


Figure.S3 Flow cytometric gating strategy for phagocytosis efficiency.


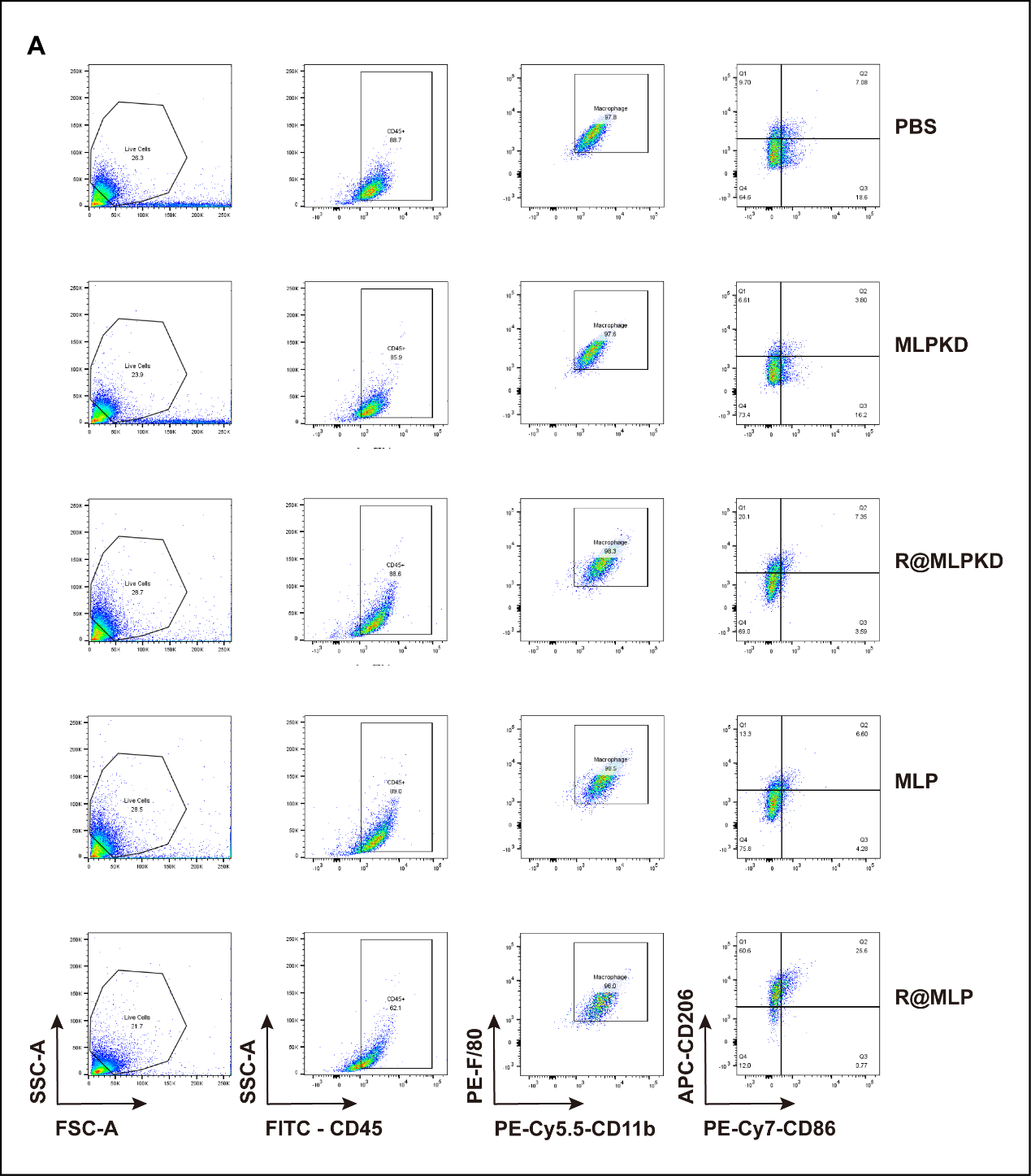


Figure.S4 Flow cytometric gating strategy for macrophage inflammatory phenotypes (M1 and M2).


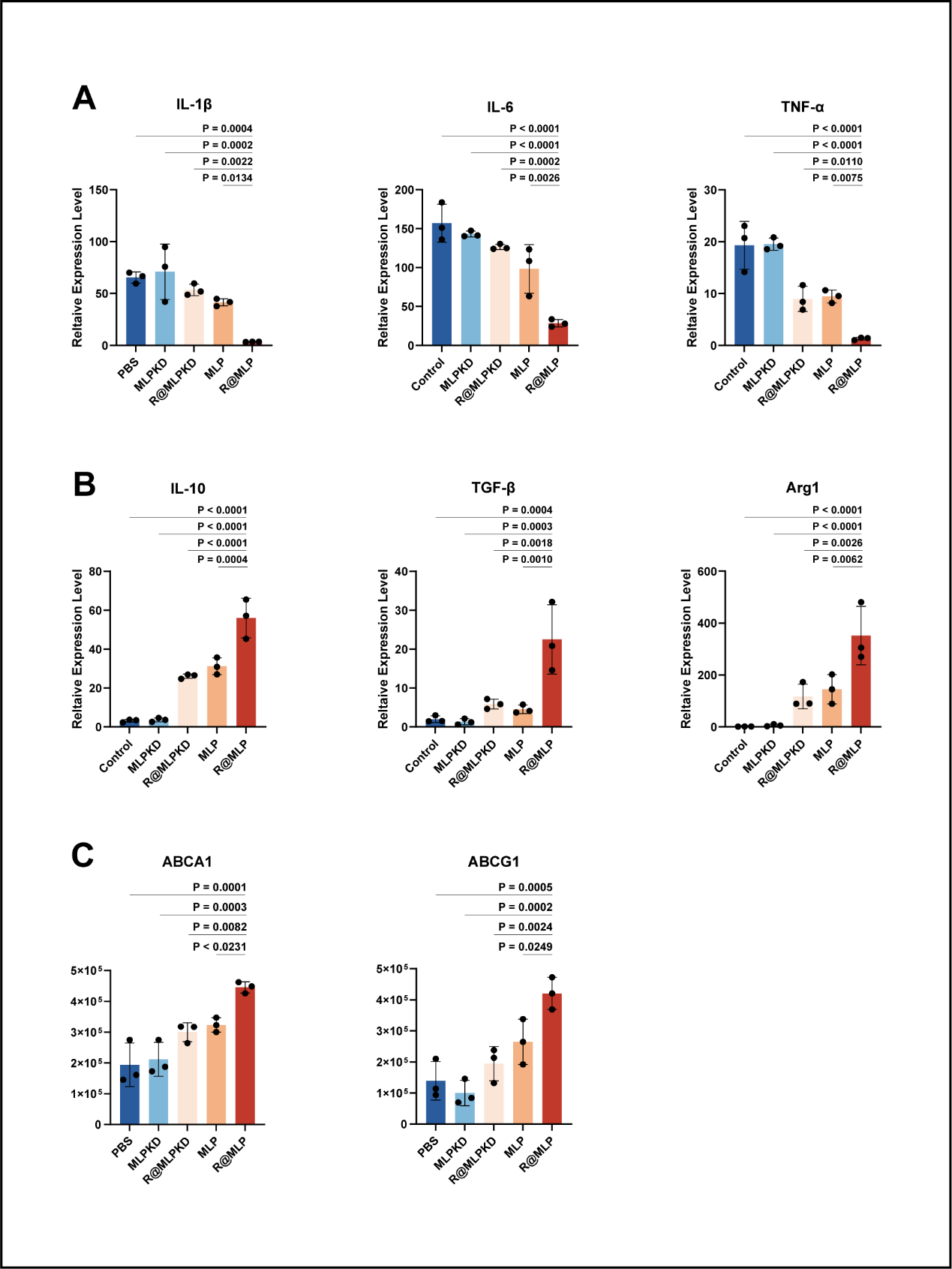


Figure.S5

(A) Quantifying IL-1β, IL-6, and TNF-α mRNA expression in macrophages by qRT-PCR(n=3). Statistical analysis was calculated using the one-way ANOVA and Tukey’s tests.

(B) Quantifying IL-10, TGF-β, and Arg-1 mRNA expression in macrophages by qRT-PCR(n=3). Statistical analysis was calculated using the one-way ANOVA and Tukey’s tests.

(C) Quantification of Western blot analysis for ABCA1 and ABCG1(n=3). Statistical analysis was calculated using the one-way ANOVA and Tukey’s tests.


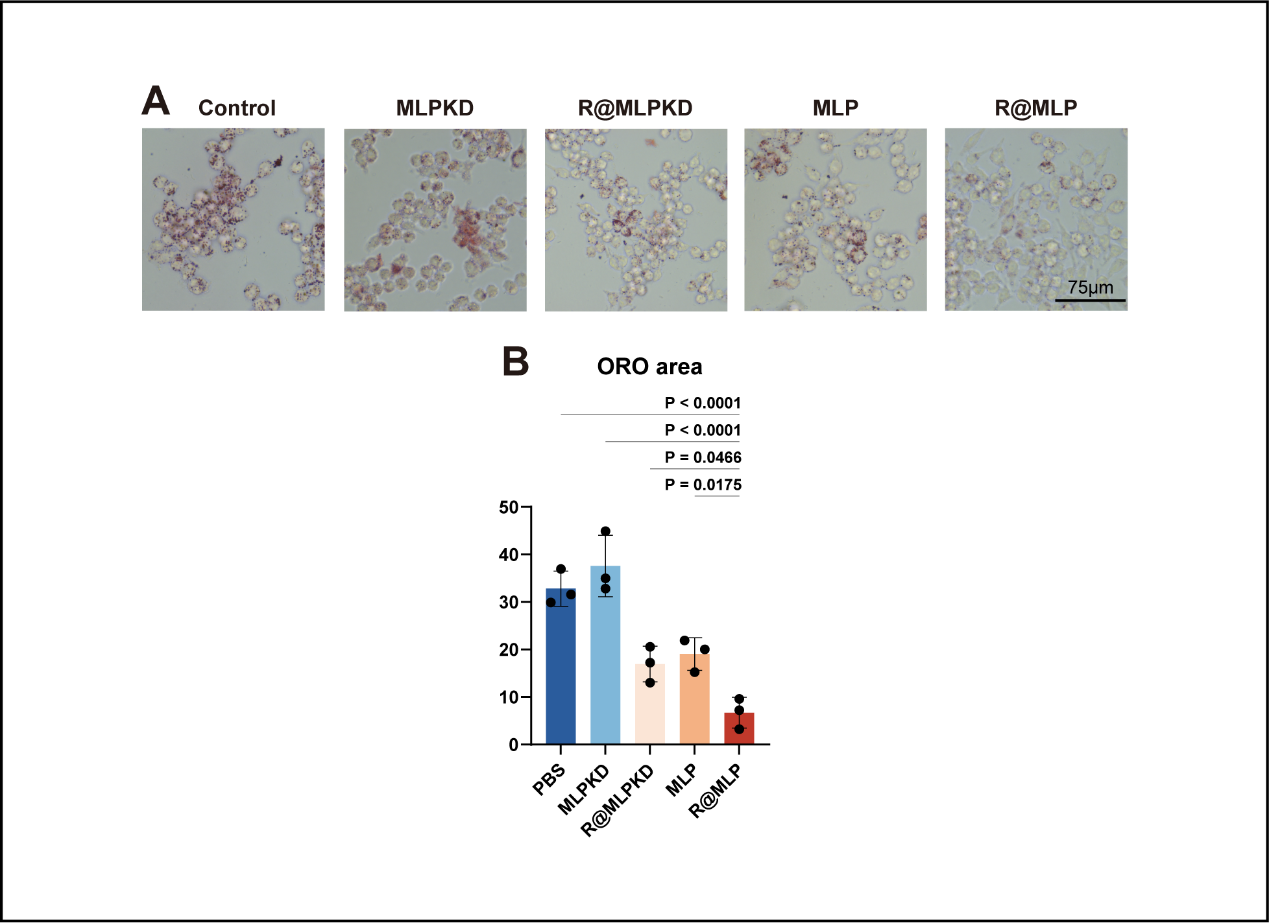


Figure.S6

(A) Intracellular lipid droplets in macrophages after phagocytosis, visualized by Oil Red O staining.

(B) Quantitative analysis of Oil Red O staining area in macrophages.


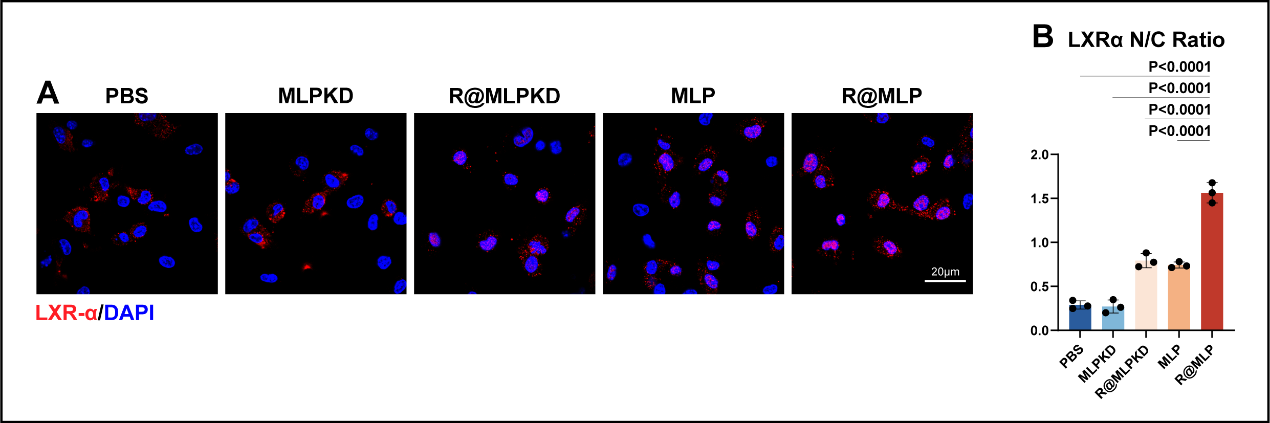


Figure.S7

(A) CLSM images of LXR-α in BMDMs co-incubated with NPs-decorated FCDACs.

(B) Quantitative analysis of LXR-α fluorescence intensity in the nucleus compared to the cytoplasm(n=3). Statistical analysis was calculated using the one-way ANOVA and Tukey’s tests.


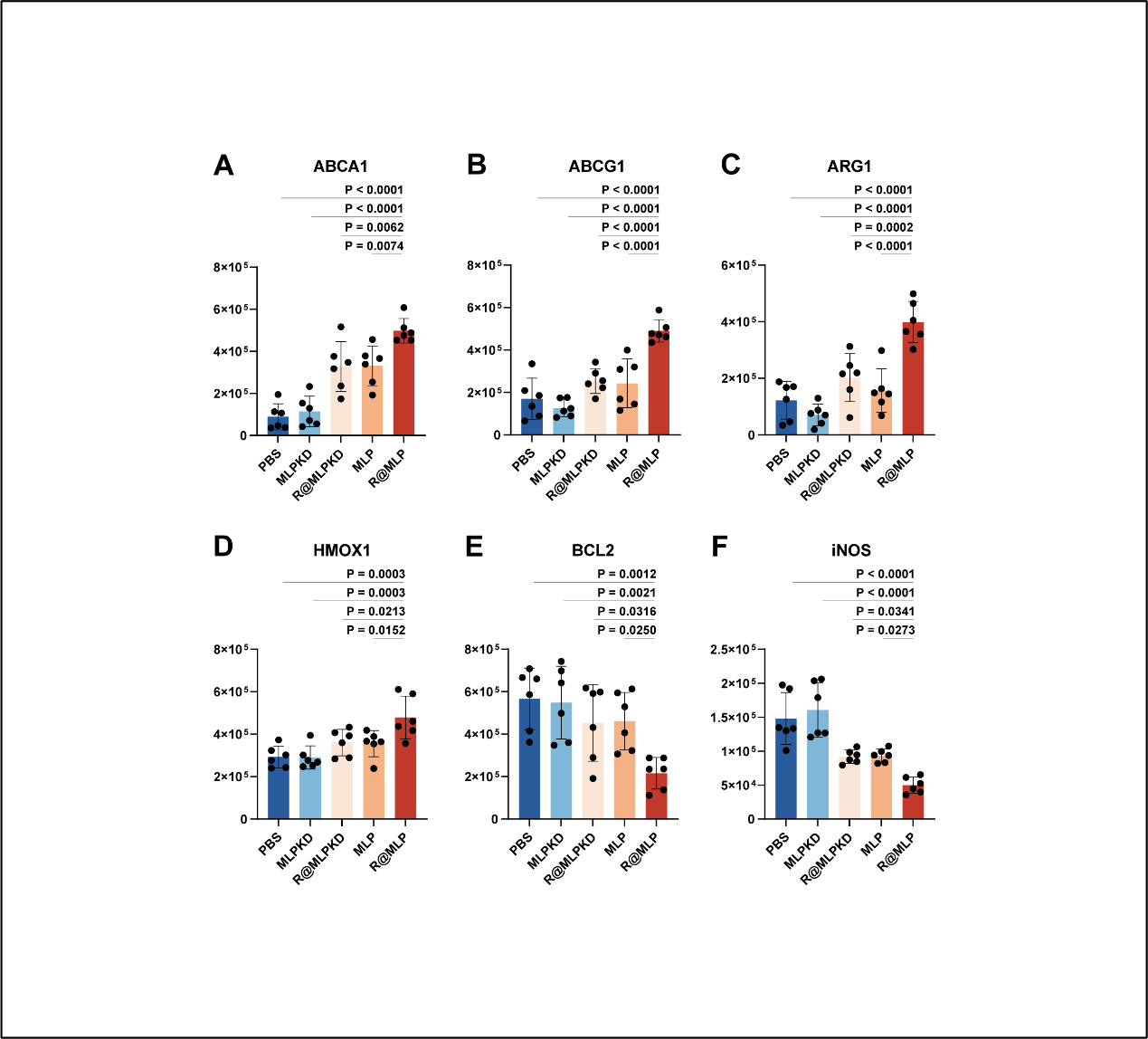


Figure.S8

Quantification of Western blot analysis for ABCA1, ABCG1, ARG1, HMOX1, BCL2, and iNOS(n=6). Statistical analysis was calculated using the one-way ANOVA and Tukey’s tests.


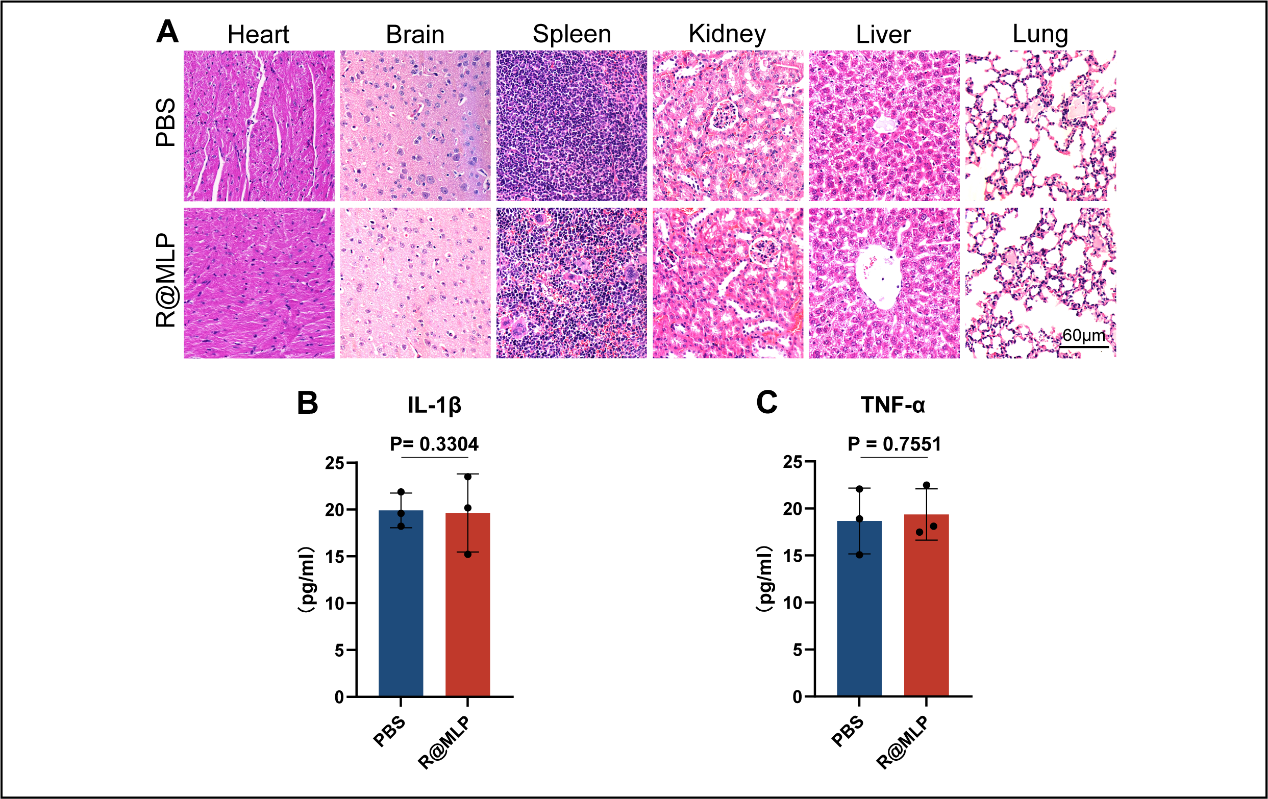


Figure.S9

(A) H&E staining of major organs of ApoE^−/−^ mice treated with different nanoparticles for 8 weeks (magnification=200 ×).

(B) and (C) IL-1β and TNF-α concentration in plasma 8 weeks after PBS or R@MLP injection(n=3). Statistical analysis was calculated using the one-way ANOVA and Tukey’s tests.
